# A Rare Presentation of Lisegang Rings in Adrenal Cavernous Hemangioma : Case Report and Literature Review

**DOI:** 10.1155/2021/9998729

**Published:** 2021-08-03

**Authors:** Wejdan Almotairi, Abdullah Alhamam, Aali Alotaibi, Tarek El Sharkawy, Hind S. Alsaif, Abdulatif Almousa, Ossamah Alsowayan, Hamed Eldarawany, Kamel Fadaak

**Affiliations:** ^1^Department of Pathology, College of Medicine, King Fahd Hospital of the University, Imam Abdulrahman Bin Faisal University Dammam, Dammam, Saudi Arabia; ^2^Department of Urology, College of Medicine, King Fahd Hospital of the University, Imam Abdulrahman Bin Faisal University, Dammam, Saudi Arabia; ^3^Department of Radiology, College of Medicine, King Fahd Hospital of the University, Imam Abdulrahman Bin Faisal University, Dammam, Saudi Arabia; ^4^Department of Pathology, College of Medicine, King Faisal University, Alhofuf, Saudi Arabia

## Abstract

**Background:**

Adrenal cavernous hemangiomas (AH) are benign nonfunctional vascular tumors rarely discovered as incidental findings on imaging studies or autopsies. This study presents a single case report of AH with another rare finding of the Liesegang ring. Also, we reviewed 73 case reports of cavernous adrenal hemangioma to provide an overview of AH's clinical characteristics. *Case Report*. A nonfunctional AH was incidentally discovered in a 59-year-old morbidly obese female patient with a 10-year history of hypertension and thyroidectomy. An abdominal computed tomography (CT) scan showed a left suprarenal mass of ∼16 cm in diameter. While the patient had no clinical manifestations from the hemangioma, all laboratory tests were within the normal values with no indication of a functional adrenal tumor. The mass was removed by open left adrenalectomy. The microscopic histological examination revealed a laminated structure with wide blood-filled spaces with a central core of necrotic and hemorrhagic changes, characteristic of a cavernous AH with the presence of a rare Liesegang ring.

**Conclusion:**

Although rare, AH should be considered as a differential diagnosis for adrenal masses. This is the first reported case of a cavernous AH with rare microscopic findings of the Liesegang ring.

## 1. Introduction

Benign vascular tumor-like growths are commonly seen in the liver and skin tissues. However, the presence of a vascular tumor, cavernous hemangioma, in the adrenal glands is a rare incidental finding often discovered on radiologic imaging (abdominal CT, MRI, or ultrasonography) or autopsy [1]. Adrenal hemangioma (AH) is a rare benign nonfunctioning tumor developing from the vascular endothelium of the adrenal gland. The first clinical case of the adrenal cavernous hemangioma was published by Johnson and Jeppesen in 1955 [2]. This was a patient presenting with a complaint of sudden-onset hypertension and no symptoms of endocrinal origin. Despite being a rare occurrence, the potential accompanying endocrinal manifestations make it imperative to study the tumor and identify its characteristic features that may help in making a diagnosis.

Usually unilateral, AH is observed later in life (after the fifth decade) with female preponderance [3, 4]. We report a case of an incidental adrenal hemangioma with a rare microscopic finding of the Liesegang ring found on the abdominal computed tomography (CT) of a 59-year-old female patient.

There are about 73 case reports of AH available from the year 1955 to date. Evidence of Liesegang ring has been reported with fibrotic, cystic, necrosed, or inflamed tissue of the skin, conjunctiva, female genital tract, or kidney [5]. Liesegang rings are formed as a result of precipitation occurring over time in highly saturated solutions. However, to the best of our knowledge, this is the first reported case of AH with histological evidence of a Liesegang ring. Following the case report, the authors also present a brief and comprehensive review of the reported cases of AH so far.

## 2. Case Report

A 59-year-old morbidly obese lady with a history of essential hypertension for ten years and hypothyroidism was referred to the urology department because of an incidental finding of a huge left suprarenal mass on the abdominal CT scan. The patient had no other complaints or clinical signs that may be attributed to adrenal (medullary or cortical) overactivity, and the physical examination had no abnormality. Laboratory tests, including urine tests for 24-hour fractionated metanephrine, were within normal limits.

**Figure 1:**
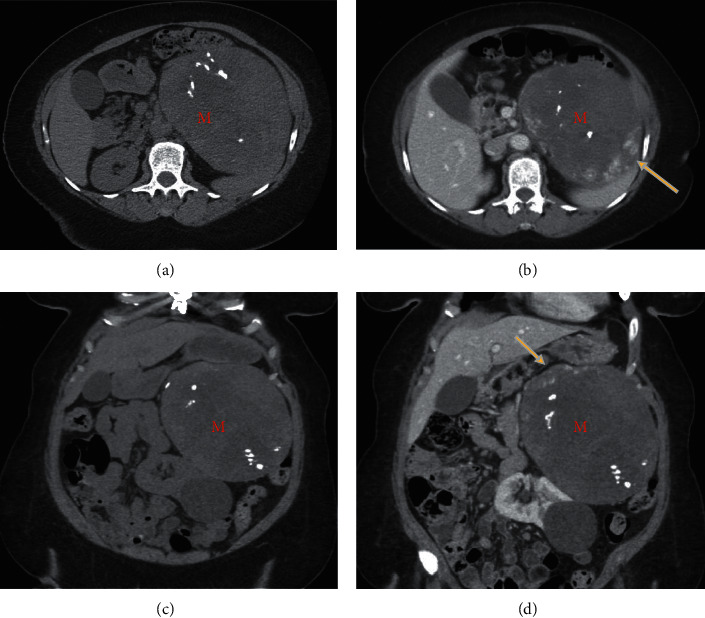
Pre- and postcontrast CT scan of the abdomen: large heterogeneous solid left adrenal mass (M) with internal calcifications and peripheral discontinuous enhancement after contrast (arrows in B and D). *Note*. Significant mass effect of the left kidney which is pushed inferiorly.

Pre- and postcontrast abdominal CT scans (Figure 1) revealed a large heterogeneous solid left adrenal gland mass with internal calcifications and central necrosis. Postcontrast showed peripheral nodular discontinuous enhancement (Figure 1; B + D). The mass caused a significant mass effect on the left kidney, which is pushed inferiorly.

**Figure 2:**
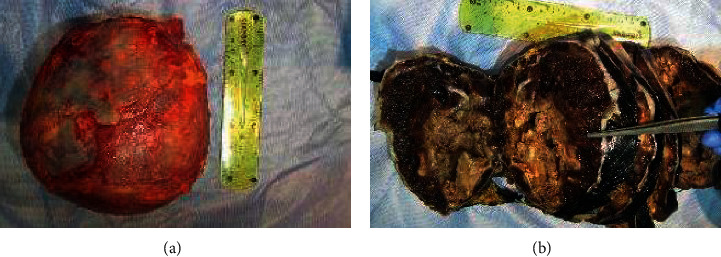
The gross examination of adrenal hemangioma (a) before and (b) after slicing, on the cut section, shows a cystic area with extensive hemorrhage.

The mass was removed through an open approach via a supraumbilical midline incision. It was well-circumscribed and has no signs of local invasion to the adjacent structures. The gross pathological examination showed an intact mass covered by a smooth and glistening capsule, measuring 16 × 15 × 10 cm and weighing 1.73 kg. On cut-section, the mass showed cystic areas with extensive hemorrhage and yellow areas, suggestive of necrosis. The residual adrenal gland could be identified (Figure 2).

**Figure 3:**
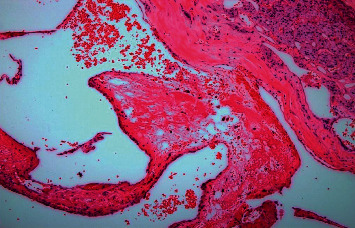
Microscopic examination of the adrenal cavernous hemangioma showing cystic dilated vascular spaces filled with blood and lined with a single layer of mature endothelium, with adjacent compressed adrenal gland (hematoxylin-eosin staining ×40).

**Figure 4:**
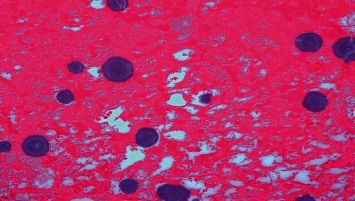
Microscopic examination showing fibrinous matrix with many basophilic, lamellar structures (the rare: Liesegang ring) (hematoxylin-eosin staining ×100).

On histological examination, the specimen consisted mainly of infarcted, necrotic tissue with cyst-like vascular spaces that were expanded due to collection of blood and were lined by a single layer of mature endothelial cells, without atypia or mitosis. The stroma showed extensive fibrinous material in which many Liesegangs rings were observed. The rings were assembled in a lamellar fashion similar to psammoma bodies or a parasite egg (commonly, kidney worm). With an amorphous material in the middle, the central core was surrounded by a two-layered wall that stained negative with PAS and von Kossa (for calcium). The annular structure was nonbirefringent when viewed under polarized-light microscopy, and on immunohistochemistry, the cystic lining tested positive for CD34 and CD31 (Figures 3 and 4).

The excision surgery was uneventful with no postoperative complications. The patient stayed in the hospital for three days postoperatively without any untoward incident. The six-month follow-up CT scan of the abdomen and pelvis showed no signs of any distant metastasis or recurrence.

## 3. Discussion

Due to the rarity of adrenal hemangioma, this benign form of a vascular tumor requires a definitive preoperative protocol for diagnosis since the tumor is nonfunctional and has no clinical manifestations of its own. The differential diagnoses for AH include benign adrenal, adenomas, myelolipomas, angiomyolipomas, or rare teratomas. Also, malignant adrenal neoplasms such as pheochromocytoma and adrenal cortical carcinoma may be considered while diagnosing [6]. AHs are mainly unilateral and more than 10 cm in diameter, predominantly affecting females in their fifth to a seventh decade [3]. Biochemical tests, like the 24-hour catecholamine secretion in urine, are essential to rule out any endocrine neoplasms, especially pheochromocytoma. All laboratory test results of the patient reported in this study were unremarkable.

Based on the provisional diagnosis of a malignant adrenal cortical carcinoma, surgical resection of the tumor was done as per the mandatory protocol [3, 4]. As reported above, the microscopic examination of the specimen revealed typical blood-filled dilated spaces of vascular origin with a monolayer endothelium. AH is usually a cavernous type (big vessels with wide blood-filled spaces within) [3] and rarely capillary in type (small well-defined tightly packed capillaries). Also, the presence of the endothelial markers CD34 (a marker of hematopoietic progenitor cells) and CD31 (a marker that stains small and large vessels in the cystic lining) confirms the vascular origin of tumors. These findings also corroborate the proposed congenital nature of AH, i.e., the influence of hereditary factors leading to vascular ectasia [3].

Another interesting finding in our study was the evidence of rare microscopic Liesegang rings seen in the AH specimen. Liesegang rings are concentric acellular structures common in chemical reactions where precipitation occurs as a consequence of the accumulation of sub- and supersaturation of insoluble components of a colloidal matrix. Not commonly seen in vivo, these rings are indicative of continuous deposition of organic components in tissues that have undergone degeneration either due to inflammation, fibrosis, necrosis, or cystic degeneration, i.e., the pathogenesis of the Liesegang ring remains unclear. Hitherto, the reported cases of Liesegang rings reveal the commonest site to be in the renal tissue, while other clinical accounts have documented these rings in the paranasal sinuses, eyelid, conjunctivae, and intraperitoneum endometriosis [5, 7, 8]. Also, isolated cases of Liesegang rings have been published in association with mammary duct ectasia and breast cyst [9, 10].

Often varying in shape, the diameter of these rings ranges from 5 to 820 nm depending on the size and location [11]. Morphologically, these structures bear a resemblance to psammoma bodies. Also, they typically test positive for PAS, highlighting the double-layered wall. However, the case presented in the current study tested negative for PAS, similar to another previously published case report [11]. The nonbirefringent nature of these rings under polarized-light microscopy was also seen in our patient's specimen.

Furthermore, the Liesegang rings seen in our study were accompanied by both necrotic and hemorrhagic changes [9, 12–16]. The reason for these changes within the hemangioma and the specific components of the Liesegang ring is yet to be determined. However, the immunohistochemical and histochemical staining for iron (Prussian blue), calcium (von Kossa), amyloid, mucopolysaccharide, keratin, glycogen, and hemangioma's epithelial antigen is negative. Additionally, using special stains, radiographic imaging, and scanning-electron microscopy, it has been demonstrated that, at times, these rings contain iron, silicon, and sulfur. Also, Tuur et al. have reported the presence of Ca++ ions, organic polycations and polyanions, and other inorganic anions in these rings [5]. Because of this chemical composition, these rings have often been misidentified for parasites' ova, green growth, calcific deposits, corpora amylacea, psammoma bodies, and the spheroid kind of amyloid. Therefore, even though these rings are a rare finding, it is imperative to characteristically study them to avoid misidentification with parasital ova. The typical presentation of a lamellar structure with a double-layered wall, cross-striations flowing radially, may be used for precise diagnosis [12, 13, 15, 17–19].

Besides, the characteristic presence of peripheral discontinuous nodular enhancement with a centripetal pattern on the contrast-enhanced CT (CECT) is highly suggestive of AH [20, 21]. Following diagnosis, the standardized treatment for an enlarging symptomatic adrenal tumor or suspected carcinoma is surgical resection, irrespective of its endocrine activity [4].

This review aimed to identify and systematically present the typical clinical features of the cavernous adrenal hemangioma to aid in diagnosis and treatment planning. Approximately 73 cases of cavernous AH have been published from 1955 through 2020 which were reviewed in the present study (Table 1). The average age at which the patients were diagnosed was 55 years (range: 19–84 years old), and the prevalence of female gender in these patients was approximately 1.7 times more than the male gender [22]. Generally unilateral with no side preference in most cases, only one case of bilateral involvement has been reported so far [21]. The usual size has been reported to be above 10 cm in diameter [1] ranging up to 35 cm [23]. Endocrinology examinations of the adrenal tumor were normal for 67 out of 73 cases. Of the six clinically functional adrenal hemangiomas identified, three cases were of hyperaldosteronism, while the other three had subclinical Cushing's syndrome [24]. The mean diameter of all these reported cases of adrenal hemangiomas with documented specific tumor characteristics was 11 cm and weighed an average of 752 gm [24].

Of the 73 cases reviewed, 42 (57.5%) were discovered incidentally with no clinical symptoms, while the other 31 (42.5%) had clinical manifestations, including abdominal symptoms of epigastric pain and heaviness (*n* = 10) and pain or discomfort in the flank (*n* = 8), and 3 patients presented with rupture of the adrenal mass leading to retroperitoneal hemorrhage and hematoma [25–27].

Surgical mass excision was done in 69 cases, of which 16 patients (22.5%) had laparoscopic surgery. Of the two nonoperated patients, one patient died from an unrelated cause, while the other opted for monitoring and observation since the tumor size was small and no clinical manifestations were seen [22, 24].

Lastly, mitotane, an established adrenolytic drug, was used in these patients that did not affect the viability of primary culture cells. However, cytotoxic drugs like doxorubicin decreased the viability of these cells. Furthermore, there is evidence that sunitinib, a multitarget tyrosine kinase inhibitor, drastically reduced the viability of the primary culture cells, indicating that the drug and its analogs may be useful in medically treating adrenal hemangiomas [4].

## 4. Conclusion

Adrenal hemangioma should be considered as an important differential diagnosis of adrenal neoplasms despite being a rare finding. The treatment of choice remains surgical excision owing to the possibility of malignancy.

## Figures and Tables

**Table 1:** Summary of the adrenal cavernous hemangiomas reported between 1955 and 2020.

Characteristics	Data (*N* = 71)^*∗*^
The median age in years (range)	55 (19–84)

*Sex*	

Male : female	31 (42.5%): 42(57.5%)

*Site*	

Right : left	37 (50.7%): 36 (49.3%)
Mean size of the mass in centimeters^*∗∗*^	11

*Clinical presentation: no. (%)*	

Incidental findings	42 (57.5%)
Symptomatic	31 (42.5%)
Flank pain or discomfort	8 (10.9%)
Abdominal symptoms	10 (13.7%)
Retroperitoneal hemorrhage	3 (4.1%)
Others	10 (13.7%)

*Metabolic workup: no. (%)*	

Normal	67 (92%)
Abnormal	6 (8%)
Hyperaldosteronism	3 (4%)
Subclinical Cushing's syndrome	3(4%)

^*∗*^The percentage was calculated based on the total number of cases in which the mass's surgical removal was done (*n* = 71). ^*∗∗*^Size was determined by gross pathological examination.

## Data Availability

All data used to support the findings of this study are available from the corresponding author upon request.
